# Divergent Evolution of E1A CR3 in Human Adenovirus Species D

**DOI:** 10.3390/v11020143

**Published:** 2019-02-08

**Authors:** Gurdeep Singh, Ashrafali M. Ismail, Jeong Yoon Lee, Mirja Ramke, Ji Sun Lee, David W. Dyer, Donald Seto, Jaya Rajaiya, James Chodosh

**Affiliations:** 1 Department of Ophthalmology, Howe Laboratory, Massachusetts Eye and Ear Infirmary, Harvard Medical School, Boston, MA 02114, USA; gur.singh@mail.utoronto.ca (G.S.); Mohamed_Ismail@meei.harvard.edu (A.M.I.); jeongyoon.lee@jbnu.ac.kr (J.Y.L.); mirja.ramke@gmx.de (M.R.); ljs02122013@gmail.com (J.S.L.); 2 Department of Microbiology and Immunology, University of Oklahoma Health Sciences Center, Oklahoma City, OK 73104, USA; david-dyer@ouhsc.edu; 3 Bioinformatics and Computational Biology Program, School of Systems Biology, George Mason University, Manassas, VA 22030, USA; dseto@gmu.edu

**Keywords:** adenovirus, E1A, viral evolution

## Abstract

Adenovirus E1A is the first viral protein expressed during infection. E1A controls critical aspects of downstream viral gene expression and cell cycle deregulation, and its function is thought to be highly conserved among adenoviruses. Various bioinformatics analyses of E1A from 38 human adenoviruses of species D (HAdV-D), including likelihood clade model partitioning, provided highly significant evidence of divergence of HAdV-Ds into two distinct groups for the conserved region 3 (CR3), present only in the E1A 13S isoform. This variance within E1A 13S of HAdV-Ds was not found in any other human adenovirus (HAdV) species. By protein sequence and structural analysis, the zinc finger motif of E1A CR3, previously shown as critical for transcriptional activation, showed the greatest differences. Subsequent codon usage bias analysis revealed substantial divergence in E1A 13S between the two groups of HAdV-Ds, suggesting that these two sub-groups of HAdV-D evolved under different cellular conditions. Hence, HAdV-D E1A embodies a previously unappreciated evolutionary divergence among HAdVs.

## 1. Introduction

Human adenoviruses (HAdVs) belong to the genus *Mastadenovirus* within the *Adenoviridae* family, and comprise seven species, HAdV-A to HAdV-G. HAdV-D contains the largest number of viruses presently recognized in GenBank, totaling 61 out of 90 HAdV genotypes. Moreover, HAdV-D is the fastest growing clade with 19 of the 28 most recently emerged genotypes [1,2,3,4]. We previously identified homologous recombination as the main evolutionary force driving this rapid expansion [5,6,7,8,9]. Recombination pressure was found to be most prominent in the major structural components of the viral capsid, specifically the penton base, hexon, and fiber proteins, along with the three E3 CR1 open reading frames (ORFs). These regions are also the most hypervariable among all the ORFs in the HAdV-D genome. These insights were possible only through continued efforts to fully characterize newly identified adenoviruses through whole-genome sequencing [5,10]. The relative abundance of HAdV whole genome sequences now available permits the investigation of novel evolutionary mechanisms adopted by HAdV pathogens during their ontogeny. 

Adenoviral E1A is notable for several reasons. Although the protein has not been crystallized, E1A is perhaps the best-characterized adenovirus protein. The *E1A* gene is the first gene expressed during acute infection, and E1A is historically important to our understanding of adenoviral transcriptional activation, the cell cycle, and cell transformation [11,12,13]. Deletion of E1A renders the virus transcriptionally inactive and incapable of viral replication. Two major E1A transcripts, with sedimentation coefficients of 12S and 13S, are encoded from the E1A ORF by differential splicing [14] and expressed as early as one hour after infection. These two isoforms share three out of four domains, comprising conserved regions (CR) 1, 2, and 4 [15,16,17], which are conserved among all human and simian adenoviruses (SAdVs). The two E1A isoforms differ by the presence of conserved region 3 (CR3) in the larger E1A 13S and its absence in the 12S transcript. CR3 is critically important to transcriptional activation of other early viral genes [18,19]. However, most prior studies on E1A CR3 were performed only with viruses from HAdV-C (specifically types 2 and 5), in which CR3 is relatively truncated compared to that of other HAdV species: 46 amino acids (aa) in length as compared to, for example, 62 aa in HAdV-D. Beyond one notable study by Ablack and coworkers, comparing the transcriptional activation of E1A CR3 from representative viruses of six HAdV species [20], including HAdV-D9 as the representative for HAdV-D, relatively little is known about the E1A CR3 of those HAdVs that are not within species C. 

A major sub-domain described in HAdV-C5 CR3 is the N-terminal zinc finger region, believed to be critical for transcriptional activation. In E1A transactivation, this sub-domain interacts with the mediator complex subunit 23 (MED23) protein and possibly with the TATA-binding protein (TBP), the latter part of the transcription factor II D (TFIID) complex [21,22,23,24,25]. Another important functional CR3 sub-domain is the C-terminal promoter targeting region which binds to TBP-associated factors (TAFs) [26,27] and transcription factors, including OCT-4 and ATF-2 [28,29], whose binding sites are found in the adenoviral early gene promoter regions. CR3 also binds independently to p300 for transcriptional activation [30]. These functions are balanced to some degree by E1A CR1, which interacts with transcriptional co-activators such as the CREB-binding protein (CBP) and p300 to repress their activity in host gene expression [31,32]. CR1 and CR2 both participate in the binding of E1A to the tumor suppressor retinoblastoma protein (pRb) to displace E2F transcription factors, resulting in S-phase entry [33,34,35]. The C-terminal region of E1A, containing CR4, binds to E1A C-terminal binding protein (CtBP) to repress host transcription through histone modifications [36].

CR1–CR4 are present in the *E1A* ORF of all known HAdVs [16], but it has been shown previously that the impact of E1A on the infected host can be HAdV-species and type-dependent, further suggesting that a detailed examination of E1A in HAdV species other than HAdV-C is warranted. For example, HAdV-A12 E1A is oncogenic in hamsters and down-regulates MHC class I in rat transformed cells, while HAdV-C5 E1A has no similar activity [37,38,39]. Conservation of putatively critical amino acids in CR regions in multiple species has been studied [17], but the ontogeny of differences between or within species has not. We previously found that proteotyping, a novel bioinformatics tool used to elucidate amino acid signatures, [5,40] was invaluable for understanding intra-species conservation within hypervariable E3 regions of HAdV-D [6], resulting in the identification of unique proteotypes having potentially unique functional features [41]. In the current study, we applied proteotyping along with classical genomic and structural studies to demonstrate two unique subgroupings within the HAdV-D E1A 13S transcript, with evolutionary significance.

## 2. Materials and Methods 

### 2.1. Sequence Analysis 

*E1A* sequences from HAdVs representing species A–G used in this study were retrieved from NCBI (https://www.ncbi.nlm.nih.gov/nucleotide/), and aligned using MAFFT [42] and Clustal W in MEGA6.06 [43] using default parameters. Phylogenetic trees were generated using the bootstrapped neighbor-joining distance method (500 replicates) with MEGA6.06 and maximum likelihood with PhyML (HKY85 model) [44]. The PhyML generated unrooted tree in Newick format was used to label all HAdV-Ds as a single clade partition ($1) or two separate clade partitions, group 1 ($1) and group 2 ($2), while the remaining HAdVs belonging to the other 6 species (A-C, E-G) were labeled as a single clade partition ($0 as default). The corresponding labeled trees in Newick format along with *E1A* coding sequences aligned in respective preserved codon position in PHYLIP format were used as input for Clade model C (CmC) divergence selection analysis [45]. PAML 4.8 [46] CmC analysis with settings ‘Model = 3 and NSsites = 2’ was used to identify divergence selection acting on two clades separating HAdV-Ds into two classes. Using codeml, an alternate model was represented with a phylogenetic tree having each class as separate clades (complex tree) and tested against the tree with all HAdV-Ds in a single clade (simpler tree) as a null model to identify divergence. A likelihood ratio test (LRT) between the models was calculated using the resulting ln-likelihood (ln L) for null and alternate models. The statistical significance was calculated from the LRT value and difference in parameter number using a χ2 distribution [47].

### 2.2. Proteotyping 

Proteotyping was performed as previously described [5,6]. Amino acid sequences were aligned using ClustalW, and then transferred to MEGA to generate maximum likelihood trees. E1A sequences were then rearranged in the multi-sequence alignment in the same order as in the phylogenetic tree. For each amino acid position, the consensus residues were identified and residues matching the consensus at each respective position colored white. Gaps in the alignment were colored black. The remaining amino acids were each assigned a unique color for all occurrences. A unique proteotype was defined as a pattern representing 10% residue divergence from the consensus sequence [5,6].

### 2.3. Protein Sequence and Structural Analysis 

Multi-sequence amino acid alignment of E1A CR3 regions representing 38 HAdV-D E1A sequences was performed using MAFFT [42]. A protein model for HAdV-D37 and D56 CR3 regions was constructed using QUARK [48] (http://zhanglab.ccmb.med.umich.edu/QUARK), an ab initio protein folding and protein structure prediction algorithm, using atomic-level knowledge-based force field and Monte Carlo simulations to generate 3D protein structures in the absence of a template. The generated models were evaluated by Ramachandran plot using RAMPAGE (http://mordred.bioc.cam.ac.uk/~rapper/rampage.php/). At least 95% of residues in both models were found in either most-favored or allowed regions. Protein structure analysis and visualization were performed with UCSF Chimera software (http://www.cgl.ucsf.edu/chimera). CR3 protein structure backbone dynamics, including flexibility, and ordered and disordered regions, were predicted using DynaMine [49] (http://dynamine.ibsquare.be/).

### 2.4. Codon Usage Bias Analysis 

Relative Synonymous Codon Usage (RSCU) values represent the occurrence frequency of a specific codon in a sequence as compared to other synonymous codons coding for the same amino acid under equal codon usage bias. RSCU values were calculated using DnaSP v5 [50] for all individual codons except for three stop codons, methionine, and tryptophan. The RSCU values for 38 HAdV-D sequences (62 amino acids, 59 codons) for *E1A* 13S CR3 sequence were examined by correspondence analysis (CA) in R statistical software version 3.3.2 (http://www.R-project.org/) [51], using the CA function in the FactoMineR package [52], and the first two dimensions were plotted. 

## 3. Results

### 3.1. Segregation of the 13S Isoform of HAdV-D E1A into Two Major Subclades 

**Figure 1:**
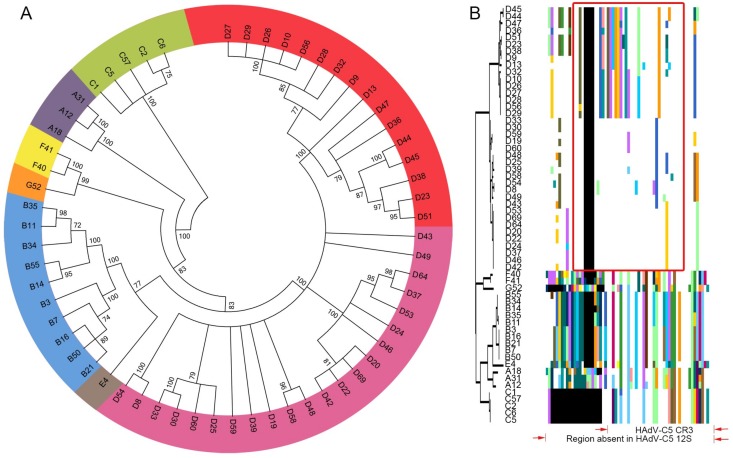
E1A 13S protein coding sequence analysis for human adenovirus (HAdV) species A–G reveals a unique evolutionary pattern for human adenoviruses of species D (HAdV-D). (**A**) Bootstrap-confirmed (500 replicates) neighbor-joining phylogenetic tree of E1A 13S protein sequence from 60 HAdVs including 38 HAdV-D. Bootstrap values below 70 were collapsed for the condensed tree. Each HAdV species is indicated by a unique color, except HAdV-D species which had two divergent subclades consisting of 16 (red) and 22 (purple) viruses. (**B**) Proteotyping analysis for the conserved region 3 (CR3) in E1A 13S protein for 60 HAdVs from seven HAdV species. Here, multi-sequence alignment is shown in respect to the maximum-likelihood tree with the consensus sequence hidden as a white background, while non-consensus colored regions represent unique shared protein signatures. The red box outlining the HAdV-Ds represents the most variable region within CR3 for the two HAdV-D groups, arbitrarily named group1 (upper proteotype encompassing HAdV-D45 to D29) and group2 (lower proteotype, encompassing HAdV-D33 to D42), each with highly distinct amino acid signatures. The CR3 of HAdV-Cs, as exemplified by the well-studied HAdV-C5 (red arrows), is the smallest in length among all HAdV species.

The evolution of the E1A 13S isoform across 60 HAdVs, representing all seven species (A–G), was analyzed by phylogenomics at the protein level. As shown in Figure 1A, E1A 13S clades segregated as expected according to existing HAdV species delineations. The largest membered HAdV species, B and D, showed further subdivisions with two subclades each, containing roughly equivalent numbers of HAdVs in each subclade. To look more specifically at the CR3 region of E1A 13S, we applied proteotyping to these 60 HAdVs. In this method, a maximum likelihood tree is generated from the putative amino acid sequences of the genomic region of interest, and a unique color is assigned to each amino acid in the resultant amino acid assembly to permit visual discrimination of subgroups. As can be seen from proteotyping (Figure 1B), two distinct HAdV-D CR3 proteotypes emerge and are resolved more clearly than the HAdV-D subclades in Figure 1A. Only one CR3 proteotype was observed for each of the other HAdV species, including HAdV-B (despite it dividing into two subclades at the complete protein level). The CR3 proteotypes for HAdV-A, B, C, E, F, and G were more similar to each other than to HAdV-D. HAdV-C had the smallest E1A CR3 13S region, comprising only 46 amino acids. Compared to HAdV-C5, with the best-studied E1A protein, the CR3 region of HAdV-Ds appears to contain an additional 16 amino acids. 

**Figure 2:**
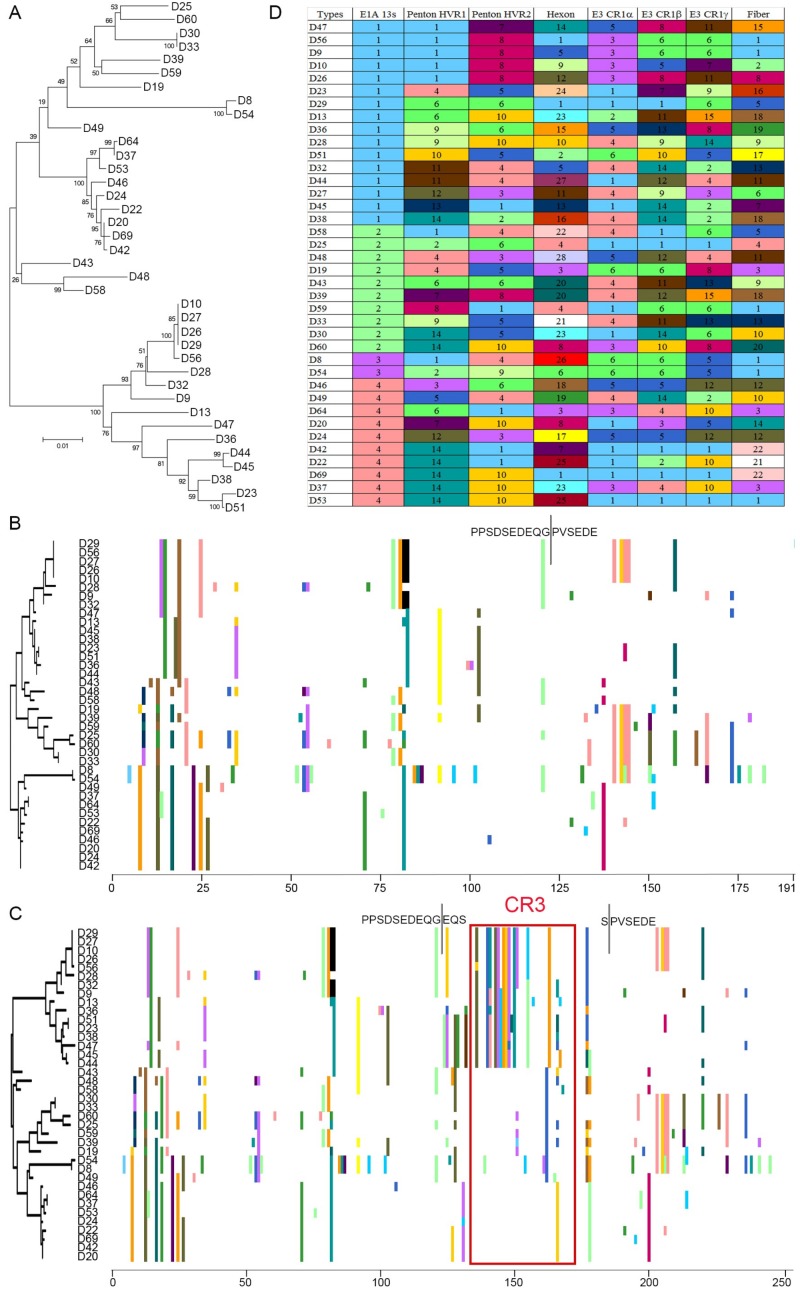
HAdV-D specific analysis of full E1A gene. (**A**) Bootstrap-confirmed (500 replicates) neighbor-joining phylogenetic tree of unspliced E1A gene nucleotide sequences from 38 HAdV-Ds. Bootstrap values below 80 are indicative of low confidence. Proteotyping analysis was performed for full coding sequences of both E1A isoforms, (**B**) 12S and (**C**) 13S, for 38 HAdV-D sequences. No significantly distinct proteotypes were observed for the E1A 12S isoform. The E1A 13S isoform clearly segregated into distinct proteotypes, arbitrarily named group1 (upper) and group2 (lower), due to differences in the CR3 region (as seen by the non-consensus protein signatures). (**D**) E1A recombination was identified in assortment analysis performed in the genomic position order of putative proteotypes for penton base hypervariable loops (HVL1 and HVL2), hexon, three E3 ORFs (CR1 α, β, and γ) and fiber open reading frames, with respect to E1A 13S proteotypes. For visual representation and understanding recombination at the whole genomic scale, each unique proteotype was assigned a number and color.

Subsequent phylogenetic analysis at the nucleotide level for the unspliced E1A ORF confirmed the clear distinction between these two subgroups within HAdV-D (Figure 2A). By proteotyping, no such division into distinct proteotypes was evident in E1A 12S (Figure 2B), nor was the origin of the difference between the two 13S subgroups apparent beyond the CR3 region (Figure 2C). Due to differential splicing of the first exon, E1A 12S in HAdV-Ds contains 191 amino acids, while E1A 13S comprises 253 amino acids. Proteotyping of HAdV-D E1A 12S suggested four proteotypes, but these were not distinctly different from one another (Figure 2B). In contrast, proteotyping of 13S showed a remarkable divergence of one proteotype (D29 to D44) from the others, with four proteotypes in total (Figure 2D). The major source of this bifurcation of E1A was the CR3 region, absent in E1A 12s transcript, and present in E1A 13S.

### 3.2. Evidence for Prior Homologous Recombination in HAdV-D E1A

HAdV-D genomes have been shown to contain seven distinguishable hypervariable regions that undergo homologous recombination as a mechanism of viral evolution, leading to the emergence of new pathogens [5,8,9]. These are the penton base hypervariable regions 1 and 2 (separated by ~450 nucleotides), hexon hypervariable regions 1–6 and 7 (closely adjacent), E3 CR1-α, E3 CR1-β, E3 CR1-γ, and fiber genes. Unlike the penton base and hexon genes, which contain hypervariable regions within otherwise highly conserved nucleotide sequence, the three E3 genes and the fiber gene are hypervariable over their entire ORFs. Hypervariable regions can recombine as independent units, or the recombination event initiating at one hypervariable region can extend to include a larger area of the genome [2,6]. As shown by proteotype analysis and subsequent sorting based on E1A 13S (Figure 2D), the data suggest that *E1A* might be an additional site of homologous recombination in HAdV-D. For example, HAdV-D47, D56, D9, D10, and D26 all share a proteotype including penton base hypervariable region (HVL)1, while the latter four viruses (D56, D9, D10, and D26) also share a proteotype with penton base HVL2. This suggests that the recombination event for HAdV-D47 *E1A* ended somewhere between penton base HVR1 and HVR2, while for the other four viruses the recombination event concluded after penton base HVR2 but before the hexon hypervariable regions, a pattern observed previously [53].

### 3.3. Evolutionary Divergence in HAdV-D E1A 13S Zinc Finger-Containing CR3 Region 

Our proteotyping and phylogenetic analyses indicated that the two separate clades in HAdV-Ds evolved divergently for the E1A-13S protein. Therefore, we tested divergence within clades and site-specific substitution constraints along entire clades at the codon level through quantifying the diversifying selection pressure acting on each clade in comparison. Clade model C [45] (CmC) in PAML [46] was used to test this hypothesis with whole E1A-13S DNA sequences for 60 HAdVs using nested models. The null model had all the HAdV-Ds labeled as a single partition or one clade (D combined), while the second partition consisted of the rest of the HAdVs belonging to other species as the background clade. Although the CR3 region might be evolving in both clades, this null model assumes no divergence within the D combined clade. The alternate model had three partitions, which included two partitions for the two subclades in HAdV-D and a third as background clade with the rest of the HAdVs belonging to other species. This model assumes the divergence between the clades in HAdV-Ds and hence is a complex version of the simpler null model. There was only one parameter difference between these models. Hence, using one degree of freedom and twice the difference in the (ln L) values between these models, the likelihood ratio test (LRT) produced a p = 6.7 × 10^−16^. This is consistent with a highly diversifying selection pressure between HAdV-D clades for the full E1A-13S (Table 1). To better understand the role of the CR3 region in this diversifying selection between the two clades, we performed another test by removing the CR3 region from the E1A-13S nucleotide sequence, which accounted for 62 amino acids. Using the same complex and simpler tree partition models as above, CmC was used to identify the divergence between the clades for this shortened sequence without CR3. Interestingly, the difference was no longer significant (*p* = 0.108), demonstrating that the divergent selection within CR3 is the major driver of divergence between the two HAdV-D E1A 13S clades (Table 1).

**Figure 3:**
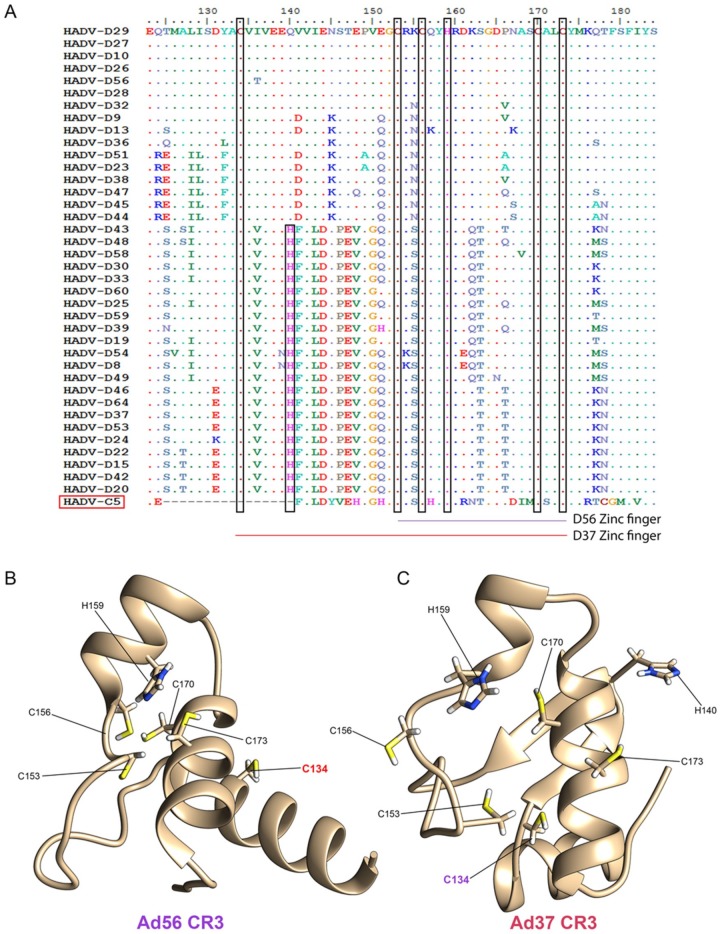
Predicted E1A CR3 zinc-finger scaffold structure in relationship to HAdV-D protein sequence divergence. (**A**) Multi-sequence alignment of 38 HAdV-Ds and the HAdV-C5 amino acid sequences representing the CR3 region, present only in the 13S E1A isoform. Conserved cysteine and histidine in the CR3 region are shown for both groups. Ab initio protein structure models for the E1A CR3 region from (**B**) HAdV-D56 and (**C**) HAdV-D37. Cysteine and histidine residues potentially involved in the zinc finger region are highlighted.

Further inspection of the HAdV-D 13S CR3 region by multi-sequence alignment revealed the area of greatest divergence between 13S sequences included the zinc finger motif responsible for transcriptional activation (Figure 3A), with an average identity of only 64.7% (55–70% range) between the two HAdV-D clades at the protein level. As previously reported [54], most of the E1A protein was intrinsically disordered except for the region of the CR3 zinc finger motif. The E1A CR3 zinc finger motif has been characterized as a Cys-4 zinc finger, comprised of four cysteine residues [55]. As seen in the multi-sequence alignment, these four cysteine residues, previously described for HAdV-C5, are also conserved in HAdV-D, and found at positions 153, 156, 170, and 173. Many zinc finger motif classes contain multiple histidine residues, and we identified a highly conserved histidine residue at position 159 in all 60 HAdVs. We also found a HAdV-D specific and conserved cysteine residue at position 134. This HAdV-D specific cysteine residue-containing region is absent in HAdV-C5, and its role is yet to be studied. Hence, our multi-sequence alignment suggested that HAdV-Ds have a novel zinc finger motif, with an extra (fifth) cysteine residue. Finally, we noted an additional histidine residue at position 140, present only in the larger of two 13S CR3 subgroups (group2) and of unknown significance. 

The structure of E1A has not been resolved. We therefore applied ab initio protein modeling to compare the impact of sequence divergence between 13S CR3 subgroups on the corresponding zinc finger fold. Models of the CR3 regions of HAdV-D56, chosen as a representative for group1, and D37, from group2, were studied as examples of each subgroup (Figure 3B,C, respectively), as they share similar tropisms [1]. In the D56 CR3 protein model, four cysteine residues formed a compact zinc finger scaffold in close proximity with the conserved histidine residue at position 159. The secondary structure of the D56 CR3 protein consists of three alpha helices, and the conserved cysteine at position 134 was distant from the core zinc binding domain. In contrast, the D37 CR3 protein model showed an alpha helix with two beta strands. The HAdV-D specific cysteine C134 in D37 was part of the zinc finger scaffold. Only the Ad37 protein model incorporated all five conserved cysteines in its scaffold, while the Ad56 model was able to incorporate only four cysteines, similar to HAdV-C5. Hence, it appears that the predicted D56 E1A zinc finger structure is similar to what was previously predicted for E1A in other HAdV species, having four cysteine residues in the zinc finger, while the D37 E1A proteotype evolved to include a fifth cysteine residue in its zinc finger scaffold structure. The ab initio models for HAdV-56 and D37, as shown in Figure 3B,C, respectively, were generated based on the entire 62 aa sequence for the E1A CR3 region. However, the great majority of studies of E1A CR3 have used HAdV-C5. HAdV-Cs are unique in that their E1A CR3 is only 46 aa in length. It could be argued that given the intrinsically disordered character of most of E1A 13S, that the extra 16 aa in other HAdV species do not contribute to the structure of their CR3 zinc finger domain. 

**Figure 4:**
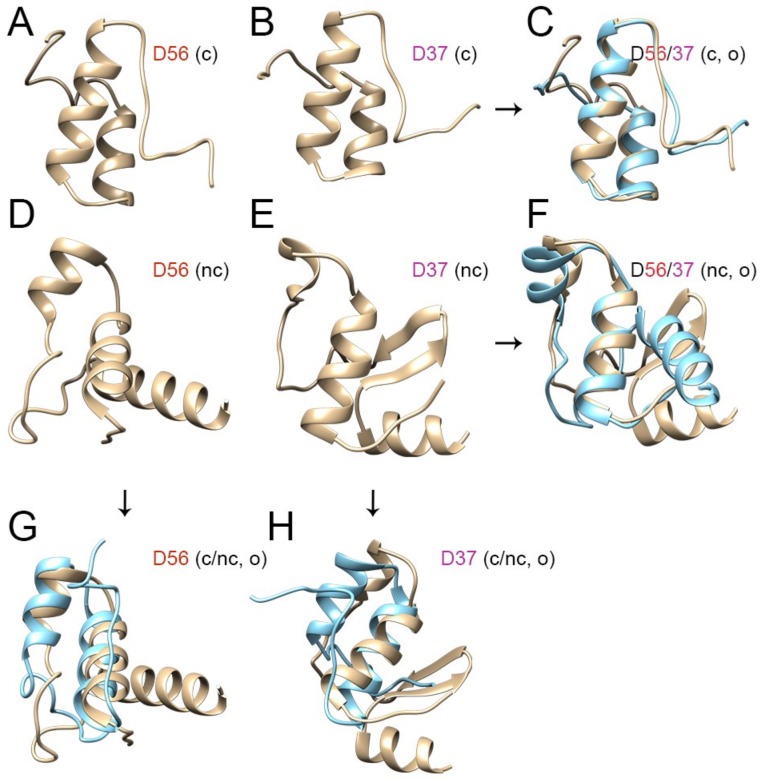
Ab initio protein structure model comparisons for E1A CR3 for HAdV-D56 and D37. The canonical (c) 46 amino acid CR3 regions for (**A**) D56 and (**B**) D37 appear very similar (**C**) when overlapped (o) (D56 in tan color and D37 in cyan). The full-length, noncanonical (nc) 62 amino acid sequences for (**D**) D56 and (**E**) D37 are more disparate (**F**). Overlapped comparison of the 46 and 62 amino acid-based models for D56 (**G**) shows relatively little difference in the structure of the zinc finger domain for D56, but a significant change in conformation of the putative zinc finger domain for D37 (**H**).

To further elucidate the effects of including the entire 62 aa in our models, we next compared ab initio models of HAdV-D56 and D37 and assessed their structural overlap using both the canonical 46 and the extended noncanonical 62 aa sequences. As shown in Figure 4, when modeling was restricted to the canonical 46 aa, the predicted structures for D56 and D37 were nearly identical (Figure 4A–C). However, when the full E1A CR3 comprising of 62 aa was modeled, the predicted structures diverged dramatically (Figure 4D–F). Including the entire 62 aa had a greater effect on the predicted structure of D37 than on D56 (Figure 4G,H). It was previously shown that ~57% of the amino acids in E1A CR3 region are required for transcriptional activation based on studies of HAdV-C5 E1A CR3 by conservative mutational analysis [17]. Moreover, the region corresponding to the first half (23 aa out of 46 aa) of the canonical E1A CR3 (of HAdV-C5) [17] in HAdV-Ds is the most divergent region between the two CR3 subgroups with only a 56% average identity. Hence, the amino acid differences between the two CR3 subgroups may be the result of evolutionary divergence in the scaffold structure while maintaining the functionality of the zinc-finger domain. 

### 3.4. Codon Usage Bias Analysis and Divergent Evolution of HAdV-D E1A 13S

**Figure 5:**
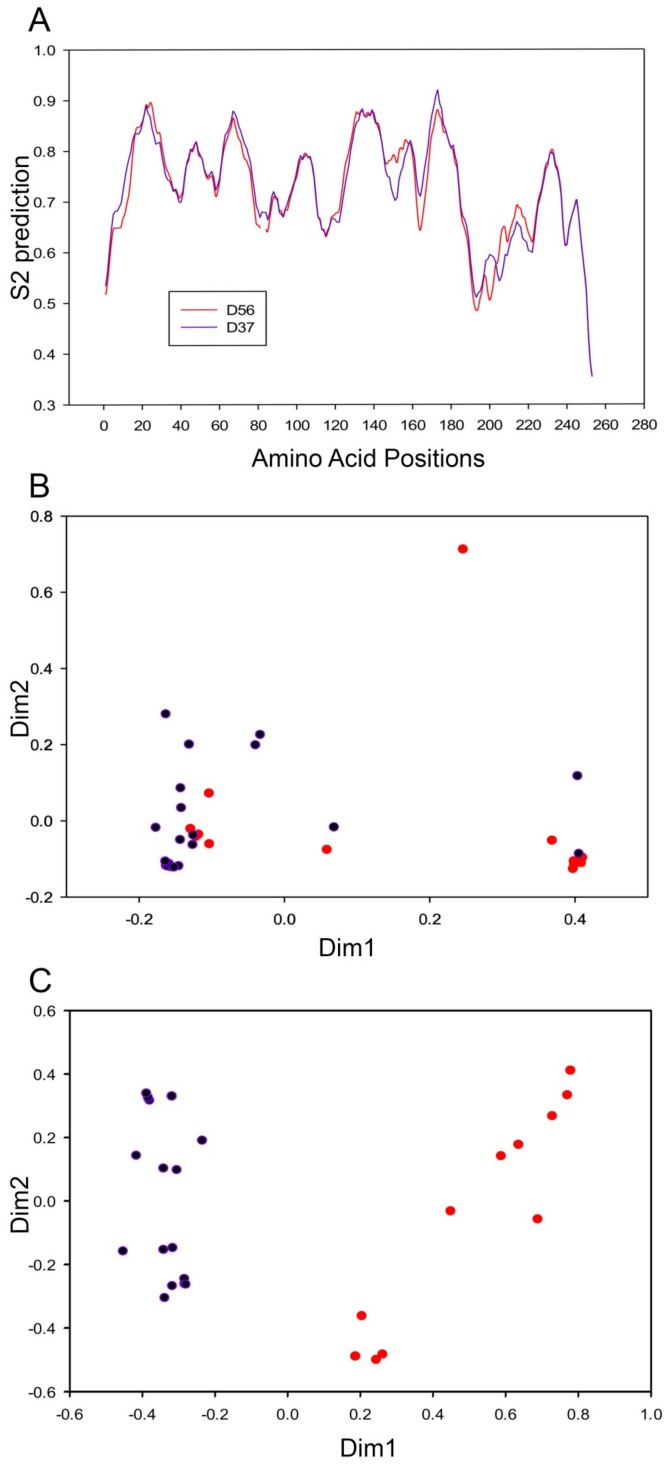
Divergence in structural flexibility and codon usage bias of the E1A protein. (**A**) DynaMine S2 predictions for E1A 13S protein flexibility and disordered regions for HAdV-D56 and HAdV-D37. Regions with S2 prediction values below 0.7 are flexible, while regions with values above 0.8 are rigid. The CR3 region is the origin for the difference in protein dynamics (at the 140–160 amino acid position) between HAdV-Ds. (**B** and **C**) Codon usage bias of 38 HAdV-Ds for 59 codons as summarized by two-dimensional plot performed with correspondence analysis for the E1A 12S isoform (**B**) and the 13S CR3 region alone (**C**). Purple color dots represent HAdV-Ds belonging to the one group, while red color dots represent the other. The difference in codon usage bias for the CR3 region indicated its evolution in different cellular environments for each group.

DynaMine analysis is a linear regression-based model trained with available NMR data to predict protein dynamics and disorder regions, and is based on amino acid sequences independent of structural modeling. DynaMine was used previously to identify interaction motifs in HAdV-C5 E1A [49]. Our analysis predicted greater structural flexibility in the region 140–160 aa, which flanks the cysteine 134 in HAdV-D37 E1A 13S relative to that of D56 (Figure 5A). Our structural predictions suggest therefore that these two CR3 groups differ principally in the zinc finger motif. We therefore employed codon usage bias analysis to examine the pathway of evolution yielding these two CR3 subgroups. RSCU values representing the frequency of occurrence of synonymous codons in a 59-dimensional codon space for each of the 38 HAdV-Ds were converged to 2-dimensional space. For the HAdV-D E1A 12S isoform, both subgroups had similar RSCU values (Figure 5B), implying that HAdV-D evolved in a similar codon-optimized environment for E1A 12S. However, the same analysis on the CR3 region of the 13S isoform revealed a clear separation between the two CR3 subgroups (Figure 5C). These data suggest that these two E1A CR3 subgroups within HAdV-D evolved in different cellular environments. 

## 4. Discussion

E1A is one of the most highly studied viral proteins, with pleiotropic influences on viral gene expression, apoptosis, and the cell cycle [13]. The E1A protein consists of four regions thought to be highly conserved among HAdV species. However, in the work presented herein, we demonstrate a unique evolutionary variance in the HAdV-D E1A CR3 region with two highly divergent CR3 subgroups. The three major capsid genes and three of eight ORFs in the E3 transcription unit of HAdV-D were previously shown to contain regions of nucleotide hypervariability with clear segregation into multiple distinct proteotypes, relative to the remainder of the highly conserved HAdV-D genome [5]. HAdV-D is the largest and most rapidly expanding HADV species [56]. Variation in nucleotide sequences coding for the three major surface capsid proteins, the hexon, penton base, and fiber, and in proteins that directly mediate immune evasion, namely E3 CR1-α, β, and γ [6], suggests successful adaptive evolution to achieve broad infectivity, replication, and viral transmission across multiple host cellular and tissue microenvironments [8,57]. The E1A CR3 region, present in the 13S but not the 12S E1A isoform, is critical to downstream viral gene expression and has a direct impact on viral replication and, therefore, on viral fitness. The ab initio modeling, in context with the other in silico analyses, suggests that the downstream transcriptional activity of E1A CR3 may differ between HAdV-D subgroups—because of differences in the amino acid backbone—which in turn would impact the motif and folding of the zinc finger scaffold critical to the binding of viral promoters for transcriptional activation. We speculate that the unique zinc-finger fold in the subgroup containing HAdV-D37, with an additional cysteine residue, might generate more robust transcriptional activation. However, whether downstream viral gene expression differs between the two HAdV-D CR3 subgroups remains to be determined by further study. Differences in the evolution (and putative function) between HAdV species have been previously shown to include marked discrepancies in size. For example, the E3 CR1β gene in HAdV-D is almost twice the length of its homolog in any other HAdV species [58]. Similarly, as noted previously, the HAdV-D E1A CR3 is larger than that reported for HAdV-C5. In the study by Ablack and colleagues comparing transcriptional activation by E1A CR3 across HAdV species, HAdV-D9 E1A CR3 transcriptional activation of a GAL4-DNA-binding domain fusion was extremely low, and well below the comparands from other HAdV species. It would be interesting to perform similar studies comparing HAdV-D9 and other members of the subgroup containing HAdV-D56, with transcriptional activation by members of the subgroup containing HAdV-D37. Further, research into the biology of E1A has contributed to the identification of many important proteins involved in mammalian gene regulation, for example, p300 and CtBP [59]. Therefore, comparing not only transcriptional activity but also the protein interactomes of HAdV-D E1A CR3 subgroups may reveal novel binding partners and transcription factor machinery components whose function is yet to be elucidated. 

Our studies of HAdV-D E1A CR3 codon usage bias indicated that both CR3 subgroups came from a common ancestor, but took different evolutionary paths to adapt to different host environments. It is unclear what specific host adaptation pressure(s) may have influenced codon usage between E1A CR3 subgroups. The close relationship between simian and human adenoviruses and prior evidence for interspecies transmission [60,61] and co-evolution [7,62,63] suggest one such possibility. Regardless, our results provide a platform to better understand zinc-finger transcription factor motif evolution in the context of viral pathogenesis, and may contribute to improvements in the design of synthetic zinc finger proteins [64].

In summary, in this study, we present the discovery and delineation of two subgroups of E1A CR3 within HAdV-D species, each with a different evolutionary history and predicted protein structure. Next-generation sequencing, genomics, and bioinformatics have made it possible to understand previously hidden aspects of adenoviral evolution. 

## Figures and Tables

**Table 1:** Clade model C (CmC) clade divergence analysis.

Full E1A-13S Nucleotide Sequence							
Model	Np	ln *L*	κ	Null	LR	df	*p*
CmC_2partition_Dcombined	123	−7622.25	2.21				
CmC_Alternate_3partition_groups1_2	124	−7589.64	1.98	CmC_Dcombined_2partition	65.22	1	6.71 × 10^−16^
**62AA CR3 sequence removed from full E1A-13S**							
**Model**	**Np**	**ln *L***	**κ**	**Null**	**LR**	**df**	***p***
CmC_2partition_Dcombined	123	−5826.58	2.06				
CmC_Alternate_3partition_groups1_2	124	−5825.29	2.05	CmC_Dcombined_2partition	2.58	1	0.108

Np = number of parameters; ln L = ln likelihood; κ = ratio of transition to transversion. LR = twice the difference in ln likelihood between complex and simpler nested models. df = degrees of freedom, equal to difference in Np between complex and simpler nested models. *p* = *p*-value.
